# Supplementary material for: Potential plasma biomarkers: miRNA-29c, miRNA-21, and miRNA-155 in clinical progression of Hepatocellular Carcinoma patients
Source: PLoS One. 2022 Feb 14;17(2):e0263298. doi: 10.1371/journal.pone.0263298 (PMC8843218; doi:10.1371/journal.pone.0263298)
Supplement: Attachment — Submitted filename: Response to Reviewers.docx [file pone.0263298.s001.docx]

**RESPONSE TO REVIEWERS**

Dear Reviewers

Thank you very much for your respond, correction, and advice due to improving our manuscript. We must respond all reviewers advised and comment one by one. The file manuscript revision was mentioned as revised manuscript with track changes, manuscript revision without track changes, and final manuscript revision.

Here, below the revision statement due to reviewer advised.

**Reviewer #1**

1. *In page 2, line 6, make correction from “The collected blood samples” to “The blood samples collected from…”. None of the preceding sentences expressly provide that blood samples were collected or whom they were collected from.*

Thank you for your advice due to sentence correction:

The blood samples were collected from 2 Province Hospitals (Dr. Sardjito Hospital and Prof. Dr. Margono Soekarjo Hospital) for HCC and the Blood Bank Donor of the Indonesian Red Cross for 36 healthy controls. These blood samples were treated as follow: …

🡪the correction on page 2 line 6-9

2. *In page 2, line 8, make correction from “and every…” to “and normalization of miRNA using…”.*

Thank you for your advice due to sentence correction:

“and every miRNA was normalized using…” was corrected to “and normalization of miRNA using…”

🡪the correction on page 2, line 11

3. *In page 2, line 12, make correction to “who were calculated”. It is assumed “HCC patients” are not the subject of the calculation*

Thank you for sentence correction.

“There were only 27 HCC patients with complete clinical variables (neutrophil to lymphocyte ratio (NLR), platelet to lymphocyte ratio (PLR), platelet count, albumin, C-reactive protein (CRP), and cholinesterase (ChE)) who were calculated for regulation miRNAs based on its fold change expression miRNA target.”

🡪The sentence “who were calculated” should be changed to “that able to analyses” (the correction on page 2, line 14)

4. *In page 2, line 17, make correction from “than healthy” to “**than that healthy control subjects”. Same for “higher than”.*

Thank you for the correction.

“The miRNAs expression of HCC subjects was lower than that healthy control subjects in miRNA-29c (down regulation 1.83-fold), higher than that healthy control subjects in miRNA 21 and miRNA-155 (up regulation, 1.74-fold; 1.55-fold) respectively.” (the correction on page 2 line 11-12 from the bottom)

5. *In page 2, line 9 from the bottom, make correction from “**count were showed” to “count showed”.*

Thank you very much for the correction.

“count were showed” was changed to “count showed” (the correction on page 2, line 9-10 from the bottom)

*6. In page 10, line 5-21, add the references (9-11 and 13,14) in the correct places.*

Thank you very much for the advice.

- We re-read the “Materials and Methods” section: Micro-RNA analyses and Real time Quantitative PCR on page 7 line 24. We add the link of specific forward primers. So the sentence is changed to “The sequence-specific forward primers based on miRBase “ (<https://www.mirbase.org/cgi-bin/>) were: (the correction on page 7, line 1-2 from the bottom)

- We make a correction on the primer sequence for miRNA 21-p (page 8 line 1): 5’TGTCGGGTAGCTTATCAGAC 3’ is changed to 5’-UAGCUUAUCAGACUGAUGUUGA-3’ (the correction on page 8, line 2)

- We add references at page 10 line 6 [11,14]; page 10 line 8 [12]; page 10 line 6 from the bottom [29]; and page 10 line 1 from the bottom [12-13]

*7. Please further elaborate on why you chose these three microRNAs for this investigation.*

Thank you for your advised.

We chose three microRNAs for our research based on some literature that mention in the introduction (page 4) and in the discussion (page 21)

🡪Introduction (page 4, line 1-15)

The dysregulation of miRNAs may affect some crucial biological processes of cells leading to tumor development by increasing proliferation, decreasing apoptosis, and enhancing the metastatic potential. The miRNA-122 is the most abundant miRNA in normal liver parenchyma, accounting for more than 70% of the total miRNA in hepatocytes [8]. The miRNA-21 is one of the first detected oncogenic miRNAs, controlling the cell cycle and tumorigenesis. Serum level of miRNA-21 was significantly higher than miRNA-155 and miRNA-365 in patients with breast cancer compared to healthy subjects. Plasma levels of miRNA-122 and miRNA-21 have strong correlations with the degree of fibrosis in HCV genotype-4 patients. Consequently, they can be considered as potential biomarkers for early detection of hepatic fibrosis. Moreover, miRNA-21 can be used as a potential biomarker for early detection of HCC combined with vascular endothelial growth factor (VEGF) and 𝛼-Fetoprotein (AFP) [9-11]. And also, the serum level of miRNA-21 is able for detecting the liver necro inflammatory activity event there was not the presence of HCC [12].

🡪Discussion (page 21, line 6-9)

The miRNA 21 (PTEN as a target gene) and miRNA 155 (RhoA and TLR as target genes) have a role in metastasis effects of HCC. Oppositely, miRNA 29c (Bcl-2, Bcl-w, and Ras as target genes) has a role in apoptosis [32]. The circulation of miRNA 21 and miRNA 122 can predict progression LC to HCC with a value of OR >1 [33].

🡪added the latest study by Jin etal,2019 Circulating microRNAs as Potential Diagnostic and Prognostic Biomarkers in Hepatocellular Carcinoma (page 21 line 14-15)

(Scientific Reports, 2019. 9:10464. https://doi.org/10.1038/s41598-019-46872-8)

Revealed that identifying 12 miRNAs that are consistently and significantly expressed at higher levels in HCC patients compared to normal health (NH) volunteers. miRNA 122-5p was one of 5 potential biomarkers in chronic HBV infection. 🡪 this reference may be added at reference no 36 (automatically after references editing of references list).

*8. Is there any report about expression of these microRNAs in liver tissue or HCC?*

There are some reports/ reviews about expression miRNAs in liver tissue of HCC:

-Heyes and Chayama, 2016 (ref no. 11), mention that direct measurement of tissue miRNAs is intrusive and inconvenient as a biomarker even though measurement of serum microRNAs is much less intrusive. The miRNA levels in the liver have been found to be correlated with serum levels for a few microRNAs. The miRNA levels in the serum can estimate miRNA activity in the liver.

- Tang etal., 2016 (ref no. 14), mention about the level of miRNA 155-3p by qRT-PCR conducted from liver tissue HCC specimen and HCC cell line. The miRNA-155 expression levels showed significant elevation in HCC tissue with an increase of 1.5-6 times compared to normal liver tissue. Overexpression of miR-155-3p and miR-155-3p inhibitor were transfected into HCC cell lines to investigate its role in HCC.

- most of article mention that minimally invasive biomarkers were more convenient than tissue biopsy with comparing results of level miRNAs between serum with tissue specimen especially in HCC cases (ref no. 9-11, 12, 13)

*9. In page 16, line 3 from the bottom, the sentence beginning from “Based on the……” is not very convincing. We cannot know how these microRNAs act form this examination as it is merely a cross-sectional study.*

Thank you for your correction. the sentence “Based on the…” is in page 15, line 3 from the bottom. The complete sentence is “Based on APASL criteria of AFP ≥200 IU/ml, this study results showed AFP might be a predictor factor of up-regulation of miRNA 29c-3p and protective factor for up-regulation of miRNA 21-5p and miRNA 155-5p.”

This study design is cross-sectional study, thus “predictor factor” must be revised to “predictive factor (OR=1,25)”; and “protective factor” must be revised to “preventive factor” (R=0.32) and insert the ORs. “…. predictive factor (OR=1.25) of up-regulation of miRNA 29c-3p and preventive factor for up-regulation of miRNA 21-5p (OR=0.32) and miRNA 155-5p (OR=0.42).”

(Page 15 line 1-4)

*10. If you have data of overall survival, this study will be more valuable and you can add Kaplan-Meier method.*

We have data of overall survival for 3-years monitoring. If we add Kaplan Meier curve,

- In the method of the study on abstract (page 2 line 9-13): “There were only 27 HCC patients with complete clinical variables (neutrophil to lymphocyte ratio (NLR), platelet to lymphocyte ratio (PLR), platelet count, albumin, C-reactive protein (CRP), and cholinesterase (ChE)) who were calculated for regulation miRNAs based on its fold change expression miRNA target”. Follow with the sentence “All 27 HCC subjects were follow-up until 3-years monitoring for their overall survival “
- added sentence can see in Abstract page 2 line 15-16
- In the Materials and Methods (page 6 line 16) add the statement “All 27 HCC subjects were follow-up until 3-years monitoring for their overall survival”
- Added sentence can see in page 6 line 16-17
- Added sentence “Survival analyses were calculated using Kaplan Meier analyses” in statistical analyses (page 9 line 2 from the bottom)
- The result of overall survival analyses (Kaplan Meier curve) was showed in figure 2 (page 19) and figure 3A-3C (page 20), and also in Discussion page 23 (line 1-7 from the bottom) and page 24 (line 1-13)

*11. In page 18, line 8, “85.18% of subjects were within large tumors >5 cm in diameter and others with multiple tumors (7%). The distribution of BCLC staging C showed that more frequent than B and D.”* *Are there any differences between tumors numbers, tumors size or BCLC staging and these microRNAs? Are* *there any statistical changes? Please discuss them.*

Thank you very much for the advised.

Based on the results on table 2, the characteristic data for subjects with HCC (27 patients), there was mistyping for frequency of multiple tumors was 19 subjects (70.37%). 🡪 the correction page 12 line 10 and table 2.

The differences between tumors numbers, tumors size or BCLC staging based on these microRNAs regulation after statistical analyses can be seen in table 6 (page 18), and the result showed no statistically significant p>0.05). The description might be seen at page 16 line 5-8 from the bottom.

*12. In page 20, line 3 from the bottom, does “AUC=0.5882” have any meaning? Please add figure of AUC.*

Thank you very much for the advised.

The meaning of AUC = 0.5882 is a 58.82% chance that the model will be unsatisfactory to distinguish between positive class and negative class. Thus, we use Tertile-1 (T1) as the cut-off value of PLR (PLR ≥ 292.40), and the result of OR was more for every miRNAs regulation. The cut-off PLR using tertile 1 (T1≥ 292.40) was more consistent than PLR ≥165.83 (cut off based on AUC analyses) in miRNAs regulation.

🡪the revision in expertise AUC and AUC figure for PLR page 15 line 9-18 and insert figure 1B of AUC curve at page 16 and add reference [31] at page 15 line 14 automatically the reference list changes

🡪The revision in expertise AUC and AUC figure for NLR cutoff ≥4 in page 15 line 1-8. Figure 1A for AUC curve at page 16

*13. In page 20, line 3 from the bottom- in page 21, line 4, it is not clear why the author included two cut-off values (i.e.165.83 and 292.40).*

Thank you for your comment.

🡪At the first calculation PLR cut off was based on AUC analyses (figure 1B), but the result PLR≥165.83 with the AUC = 0.5882 is a 58.82% chance that the model will be unsatisfactory to distinguish between positive class and negative class. Thus, we re-calculated the cut off using the Tertile category.

Reported regarding PLR in HCC patients using tertile category was published by Huang GQ etal., 2017 Stratified Platelet-to-lymphocyte Ratio: A Novel Target for Prognostic Prediction of Hepatocellular Carcinoma after Curative Liver Resection (Journal of Clinical and Translational Hepatology 2017 vol. XX | 1–8) (reference: no 41 before manuscript revision 🡪 no 31 after revision). Patients with the lowest tertile of PLR (T1) had favorable 5-year survival following surgery (58%); however, those in the tertile of PLR (T3) had poor outcomes (30%) in order to predict overall survival (OS).

🡪 we insert the word “however” in the paragraph “PLR may be a predictive factor up-regulation of miRNA 29c-3p (OR=1.6) and protective factor up-regulation of miRNA 21-5p (OR=0.65). However, if cut-off value of PLR was used the tertile 1 (T1) (PLR ≥ 292.40),…….” (page 15, line 13)

🡪 we insert the sentence “The cut-off PLR using tertile 1 (T1≥ 292.40) was more consistent than PLR ≥165.83 (cut off based on AUC analyses) in miRNAs regulation.” (page 15, line 17-18)

*14. Please add statistical value in Table 5.*

Thank you for the advised.

🡪The revised and edited of the table 5

*15. Please add details about direct targets of the three microRNAs and explanation as to miRNA-155 in the discussion part.*

🡪 Thank you very much for the advised.

The revision of the explanation about direct targets of the three miRNA was mentioned in discussion part page 21 line 6-9.

We added some sentences about the potential biomarkers of miRNA in diagnosis and prognosis of HCC (page 21 line 14-15; page 22 line 8-22)

*16. Do you have any data between clinical characteristics and matching these microRNAs?*

🡪 Thank you very much, we only analyzed the clinical data as a routine examination such as routine blood examination, liver function test, tumor marker (AFP), liver ultrasound and MSCT 3-phases examinations. Because of the low prevalence of HCC patients who come to our hospitals, we can’t match the clinical variables with these miRNAs.

*17.* *How will this report be helpful for medical treatment of LC or hepatitis patients? Please discuss.*

🡪Thank you for the advised. We added some references (ref.31; 36; 43; 45-50) related to our study that mention on the simple or routine examination in daily practice that may improve the survival prediction if combining with regulation of miRNA.

Due to some revisions, we must do for paragraph and page editing.

**Reviewer #2:**

*In this study, Ratnasari et al. evaluated the expression level of miRNA-29c, miRNA-21, and miRNA-155 in plasma of 27 HCC confirmed patients and compared them with healthy controls. The correlation study with miRNA levels and clinical progression parameters concluded that miR-29c and miR-155 could be used as potential biomarkers in HCC patients.* *Overall, the study presents limited information and defines HCC in general without dissecting the effect of causing agents on miRNA expression.* *Among the 27 HCC patients, 16 HBV and 2 were HCV-positive patients. However, it is not clear whether there are any differences at the level of the miRNA within the studied groups. Moreover**, the overall sample size is not adequate to conclude as a biomarker. Even* *within the different age groups, it is not clear how these miRNAs vary.*

Thank you very much for the respond and advised

🡪The study presents limited information and defines HCC in general without dissecting the effect of causing agents on miRNA expression.

🡪The study focused on relationship between clinical with three targeting miRNAs, without any intervention of the therapy. Reviewer ^#^1^#^ advised for increasing the clinical touched by survival analyses. Thus, the methods should be improved to retrospective study with follow-up 3 years (cohort retrospective)

🡪Among the 27 HCC patients, 16 HBV and 2 were HCV-positive patients. However, it is not clear whether there are any differences at the level of the miRNA within the studied groups.

Because of the viral load haven’t examined, so we cannot analyze the relation between viral load with miRNA regulation. However, we calculated the relation between miRNAs with the etiology (HBV, HCV and other etiology). The analyses were showed in table 6 (Analyses of HCC nodule, BCLC stagging and etiology with miRNAs regulation in 27 HCC subjects). Significant difference showed in miRNA 21-5p dan miRNA 155-5p between HBV and other, even not significant difference showed in HCV because of 2 subjects only, and zero point in 1 cell 2x2 table.

🡪 The overall sample size is not adequate to conclude as a biomarker

The three miRNAs (miRNA 29c-3p, miRNA 21-5p, and miRNA 155-5p) were choose based on the references that consistence as an oncomiR or tumor suppressor. However, small sample sized that eligible for miRNA 27 only who have clinical data from 36 HCC subjects. The subjects were recruited from 2 Hospitals only.

🡪within the different age groups, it is not clear how these miRNAs vary.

Yes, we didn’t analyze regulation miRNA and clinical marker based on age groups because of limited sample sized. The study limitations were stated about study design, sample sized, only 3 miRNAs, and no subject matching.

For more details, We attached our responses one by one into the manuscript tracking below

Thank you very much for the advises and attentions. We hope the revision statement may answer all reviewers advises and questions

Sincerely yours,

Neneng Ratnasari

**Potential plasma biomarkers: miRNA-29c, miRNA-21 and miRNA-155 in clinical progression of Hepatocellular Carcinoma patients**

Neneng Ratnasari^1*^, Puji Lestari^2¶#a^, Dede Renovaldi^2¶#b^, Juwita Raditya Ningsih^2¶#c^, Nanda Qoriansas^2¶^, Tirta Waradana^3¶^, Suharno Hakim^4&^, Nur Signa Aini Gumilas^5&^, Fahmi Indrarti^1&^, Catharina Triwikatmani^1&^, Putut Bayupurnama^1&^, Didik Setyo Heriyanto^6&^, Indwiani Astuti^7&^, Sofia Mubarika Harjana^8&^

**^1^** Gastroenterology-Hepatology Division of Internal Medicine Department Faculty of Medicine, Public Health and Nursing Universitas Gadjah Mada/ Dr. Sardjito General Hospital, Indonesia.

^2^ Graduate School of Biotechnology Universitas Gadjah Mada, Indonesia.

^3^ Department Biomedicine, School of Dentistry, Faculty of Medicine Jenderal Soedirman University, Indonesia.

^4^ Internal Medicine Department Dr. Margono Soekarjo Hospital/Faculty of Medicine Universitas Jendral Soedirman, Indonesia.

^5^ Histology Department Faculty of Medicine Universitas Jendral Soedirman, Indonesia.

^6^ Anatomic Pathology Department Faculty of Medicine, Public Health and Nursing Universitas Gadjah Mada, Indonesia.

^7^ Pharmacology and therapy Department Faculty of Medicine, Public Health and Nursing Universitas Gadjah Mada, Indonesia.

^8^ Histology and Cell Biology Department Faculty of Medicine, Public Health and Nursing Universitas Gadjah Mada, Indonesia.

* Corresponding author.

E-mail: nenengratnasari@gmail.com

^¶^These authors contributed equally to this work.

^&^These authors also contributed equally to this work.

^#a^ Department of Research & Development, Dharmais Cancer Hospital, Indonesia

^#b^ Department of Biomedical Sciences, Faculty of Medicine and Health,

Universitas Muhammadiyah Jakarta, Indonesia

^#c^ Faculty of Dentistry, Universitas Muhammadiyah Surakarta, Indonesia

**Abstract**

This study evaluated differences in clinical appearance of patients with hepatocellular carcinoma (HCC) based on plasma level and regulation of microRNAs (miRNA-29c, miRNA-21, and miRNA-155). The observational-analytical study with cross-sectional design was conducted on 36 HCC patients and 36 healthy controls. The collected blood samples were treated as follows: plasma isolation, RNA isolation, cDNA synthesis, quantification by qRT-PCR using a sequence-specific forward primer and every miRNA was normalized using housekeeping-stably miRNA-16. There were only 27 HCC patients with complete clinical variables (neutrophil to lymphocyte ratio (NLR), platelet to lymphocyte ratio (PLR), platelet count, albumin, C-reactive protein (CRP), and cholinesterase (ChE)) who were calculated for regulation miRNAs based on its fold change expression miRNA target. The miRNA plasma expression was analyzed by Bio-Rad CFX 96 Manager software to determine the cycle of quantification, followed by the calculation of expression levels using Livak's methods. Data were analyzed using STATA 11.0, with significant value of p<0.05. The miRNAs expression of HCC subjects were lower than healthy in miRNA-29c (down regulation 1.83-fold), higher than healthy in miRNA 21 and miRNA-155 (up regulation, 1.74-fold; 1.55-fold) respectively. NLR, CRP, ChE and platelet count were showed significant difference in miRNA-29c regulation; though neutrophil count showed significant difference in miRNA-21 and miRNA-155 regulation (p<0.05). Conclusion: Plasma biomarkers: miRNA-21 and miRNA-155 might be potential biomarkers as onco-miR in HCC subjects, while miRNA-29c might act as a tumor suppressor. Significant evidence was identified with clinical progression based on regulation of miRNAs, which was consistent with miRNA -29c.

**Keywords**: miRNA-29c, miRNA-21, miRNA-155, clinical progression, hepatocellular carcinoma, plasma biomarker.

**Introduction**

Hepatocellular carcinoma (HCC) is the fifth most common of the major malignant liver tumors in the world and the third leading cause of mortality related cancer. The HCC incidence is varied worldwide based on geographic variation, with 81% of HCC cases occurring in developing countries and 54% of those from China [1,2]. The majority of primary HCC (70-80%) which are mostly rare are detected at early stages, with the consequence of a decline of the mortality rate [1,2].

More than 90% of HCC cases develop in chronically inflamed liver as a result of viral hepatitis, alcohol abuse and in increasing incidence in patients with non-alcohol fatty liver disease. Most of the HCC cases in developing countries are caused by hepatitis B virus (HBV) (58.8%) and hepatitis C virus (HCV) (33.4%), with different prevalence compared with developed countries with hepatitis B virus (23.3%) and hepatitis C virus (19.9%) [3,4]. Prediction of cancer progression was revealed as 5.3-148 times higher in HBsAg positive and 20 times higher in anti-HCV positive [3,4].

There are various pathogenesis of HCC development in both molecular and histological views. The alteration of essential gene regulation of cellular processes has a role in these processes, such as cell cycle control, cell growth, apoptosis, migration and cell deployment. MicroRNAs (miRNAs) are one of the protein regulators that are associated with carcinogenesis pathway. The involvement of miRNAs in tumorigenesis and tumor progression is well established, because they can act as a tumor suppressor or promoter of oncogenesis depending on cellular function of their targets. So far, 20–80% of transcribed human genes are regulated by miRNAs which are present not only in tissue cells but also in body fluids [5-7].

The dysregulation of miRNAs may affect some crucial biological processes of cells leading to tumor development by increasing proliferation, decreasing apoptosis, and enhancing the metastatic potential. The miRNA-122 is the most abundant miRNA in normal liver parenchyma, accounting for more than 70% of the total miRNA in hepatocytes [8]. The miRNA-21 is one of the first detected oncogenic miRNAs, controlling the cell cycle and tumorigenesis. Serum level of miRNA-21 was significantly higher than miRNA-155 and miRNA-365 in patients with breast cancer compared to healthy subjects. Plasma levels of miRNA-122 and miRNA-21 have strong correlations with the degree of fibrosis in HCV genotype-4 patients. Consequently, they can be considered as potential biomarkers for early detection of hepatic fibrosis. Moreover, miRNA-21 can be used as a potential biomarker for early detection of HCC combined with vascular endothelial growth factor (VEGF) and 𝛼-Fetoprotein (AFP) [9-11]. And also, the serum level of miRNA-21 is able for detecting the liver necro inflammatory activity event there was not the presence of HCC [12].

Previous study mentioned that miRNA-21 acts as an onco-miR, while miRNA-29c and miRNA-155 act as tumor suppressor miRNA in HCC. The three miRNAs might be detected in HCC and could be used as minimally-invasive biomarkers in HCC detection. The miRNA-155 expression levels showed significant elevation in HCC tissue with an increase of 1.5-6 times compared to normal liver tissue [13,14].

Alanine aminotransferase (ALT) serum level remains a more common feature as a biomarker for liver injury dysfunction more than liver functions only. The serum AFP is the most widely used tumor biomarker currently in HCC patients even though the specificity of AFP is low [15]. However, AFP is usually used as clinical biomarker of severity of liver inflammation in patients with HCC and screening of HCC evidence [16,17].

Previous studies reported about the inflammation indexes that are useful for determining mortality and morbidity risk in HCC and Liver Cirrhosis (LC), such as neutrophil-lymphocyte ratio (NLR) and platelet-lymphocyte ratio (PLR). Elevated NLR is positively associated with the pathogenesis and progression of primary HCC caused by HBV. Elevated PLR indicated a poor prognosis for patients with primary HCC. PLR may be a reliable, easily-obtained, and low-cost biomarker with prognostic potential for primary HCC [18-20].

Cholinesterase (ChE) is an enzyme that is synthesized in the hepatocytes and released into the blood as acetyl cholinesterase in red blood cells and butyric cholinesterase in serum. Activity and synthesis of ChE would be reduced if there is any cellular liver dysfunction following with damaging of cell membrane and this is a cause of diminished reserve function. Previous study revealed that the alteration of ChE in cirrhotic liver may reflect the changing in pathophysiology of synthesis at the protein and miRNA levels, even though enzymatic activity undergoes no changes [21]. Blood level of ChE was significantly reduced in LC and HCC compared with chronic hepatitis C and healthy livers [22]. Combination between ChE blood level with severity disease using Child-Pugh score can be used as a reliable measurement of the liver reserve function [23].

C-reactive protein (CRP) is one of inflammation biomarkers which are produced in the hepatocytes. High levels of CRP reflect an inflammation process in patients with infection and also in reactive inflammation in ongoing tumor growth. Inflammatory conditions may stimulate angiogenesis and promote invasive tumor progression. CRP level can be used as a prognostic marker in LC and HCC patients [24-27]. A combination of inflammation biomarkers (CRP and NLR) can be used as a predictor of tumor response and survival [28].

**Materials and Methods**

**Study population and data collection**

This research was an analytical, observational study with cross-sectional design conducted on 36 HCC patients and 36 healthy controls. Subjects were recruited from two General Hospitals at Central Java, Indonesia. The inclusion criteria were age 18-65 years old, primary HCC diagnosis was confirmed by histology examination, and two imaging (Doppler ultrasound and 3 phase MSCT-scan abdomen). The exclusion criteria were severe infection diseases, cardiovascular disease and cancers originated from other organs. All subjects who met the criteria of study read and signed the informed consent form before the study began. There were 27 HCC subjects with complete clinical data continued for the clinical study and variable analysis of the relationship between subject characteristics with regulation of miRNAs. The 36 healthy controls were recruited from the Blood Bank Donor list of the Indonesian Red Cross.

Estimation of sample sized was calculated using online sample size calculation (www.raosoft.com/samplesize.html) based on hospital rate of HCC patients during 2010 – 2015 which was 60 patients per year, 5% margin error, 90% confidence level, and 50% response distribution.

**Micro-RNA analyses**

RNA extraction

The RNA isolation was prepared from blood plasma (6 mL blood venous was fixed by EDTA, centrifuged at 1500 rpm 10 minutes). The supernatant was transferred to a 1.5 ml micro scaping tube and then the sample code was then stored at -80ºC. Total RNA isolation was performed from 200 μl of blood plasma using miRCURY RNA Isolation Kit-Biofluid (Cat No.300112, Exiqon) according to the kit procedure.

cDNA synthesis

The total RNA isolation for miRNAs was performed using miRCURY RNA isolation Kit-Biofluid, followed by cDNA synthesis for miRNA using Universal cDNA synthesis kit ll, 8-64 rxns (Cat No.203301, Exiqon) and thermal cycler PCR (Biorad c 1000). The master mix consists of 4 μL 5x reaction buffers, 9 μL Nuclease free water, 2 μL Enzyme mix, and 1 μL Spike in (sp6), up to a total volume of 16 μL reagents. 4 μL RNA sample were added to the tube. Thermal cycler Biorad C1000 were used with the protocol 60 min at 42 ^o^C for incubation time, 5 min at 95^o^C for inactivation of reverse transcriptase enzyme, and cooled at 4^o^C.

Real time Quantitative PCR

Real time Quantitative PCR (qRT-PCR) for miRNAs was performed using Real-time qPCR (Biorad CFX 96). The sequence-specific primers were used for every miRNA target: miRNA 29c-3p, miRNA 21-5p, and miRNA 155-5p. The expression of every miRNA was normalized to housekeeping- stably expressed of miRNA-16. The sequence-specific forward primers were:

5' UAGCACCAUUUGAAAUCGGUUA 3' for miRNA 29c-3p;

5’TGTCGGGTAGCTTATCAGAC 3’ for miRNA 21-5p ;

5’-UUAAUGCUAAUCGUGUAGGGU-3’ for miRNA 155-5p; and

5’-UAGCAGCACGUAAAUAUU GGCG-3’ for miRNA-16.

Reagent for performing real time qPCR miRNA was ExiLent SYBR Green master mix, 2.5 mL (Cat No. 203402, Exiqon), all primers set (miRNA-29c-3p, miRNA-21-5p, miR-155-5p, and cDNAs) prepared before. Reaction compositions consist of 5 µl SYBR Green master mix, 1µl PCR primer mix, 4µl cDNA sample which has been diluted with a ratio of 1:40.

Real time qPCR program Biorad CFX 96 were set up as follows: Denaturation 95^o^C for 10 min, amplification 40 cycle, at 95^o^C for 10 sec, 58^o^C for 1 min ramp-rate 1,6^o^ C/s optical read and melt curve analysis.

The relative expression of the target gene was calculated by the cycle of quantification (Cq) analysis using the Livak’s method [29]. The Relative expressions of miRNA formula:

ΔCq_­_ _(miRNA_ ­­_target.HCC)_ = Cq_(miRNA target HCC)_ - Cq _(mirRNA-16 HCC)_

ΔCq_­ (miRNA_ ­­_target,healthy)_ = Cq_(miRNA target, healthy)_ - Cq _(mirRNA-16, healthy)_

ΔΔCq_­ (miRNA_ ­­_target)_ = ΔCq_(miRNA target, HCC)_ - ΔCq _(miRNA target, healthy)_

Fold change expression miRNA target = 2^-ΔΔCq^

Based on the result of fold change expression for every miRNA, all miRNAs in HCC sample were adjusted the regulation to be up-regulation or down-regulation. The sample storage, RNA isolation, and cDNA synthesis were done at the Integrated Research Laboratory, Radiopoetro Building, Faculty of Medicine Public Health and Nursing Universitas Gadjah Mada. The qRT-PCR analysis was done at the Genetic Engineering Laboratory of the Center for Biotechnology Studies Universitas Gadjah Mada.

**Clinical laboratory examination**

The automatic machine with flow-cytometer was used for blood count examination. Plasma CRP was examined using agglutination method or sandwich immunometric method. Plasma ChE was examined using colorimetric analysis. Plasma albumin was examined using a comprehensive metabolic panel. The clinical laboratory examination was conducted at the Clinical Pathology Laboratory of Dr. Sardjito General Hospital, Yogyakarta, Indonesia.

**Statistical analysis**

The expressions of miRNA 29c-3p, miRNA 21-5p, and miRNA 155-5p were analyzed by Bio-Rad CFX Manager 96 Software to obtain the cycle of quantification (Cq), melting curve, and melt peak curve values. The differences in the expression of miRNAs between the blood plasma of patients with HCC and healthy individuals were tested using Mann-Whitney test. The difference in clinical variables based on the fold change expression of every mRNAs (2^-ΔΔCq^) on 27 HCC patients was calculated using the Mann-Whitney test. Based on the cut-off regulation of every miRNA target, how far the clinical predictors may predict the regulation of miRNAs was analyzed by Odds Ratio (OR), with p<0.05 as a significant value. Data analysis was calculated using STATA 11.0.

**Results**

**The miRNAs expression level and its regulation in HCC subject and healthy control**

All 36 HCC subjects who enrolled in the study continued to examine the miRNAs expression levels (ΔmiRNA) for miRNA 29c-3p, miRNA 21-5p, and miRNA 155-5p. The sequence-specific forward primers used for selection the miRNAs targeted and miRNA-16 was used as a control (housekeeping- stably) miRNA. Based on gender distribution, there were more males (23 patients; 63.9%) than females (13 patients; 36.1%), and the median age was 53 years old (min 29; max 74). The 36 healthy control subjects have recruited the study for knowing the control expression of miRNAs levels (ΔCq _miRNAs_). The ΔCq _miRNA 155-5p_ and ΔCq _miRNA 21-5p_ in HCC patients were lower than healthy controls, but only ΔCq _miRNA 155-5p_ showed significantly different values with p<0.05. The ΔCq _miRNA 29c-3p_ in HCC patients was higher than healthy controls, even though the result was not statistically significant (p>0.05) (Table 1).

Based on Livak’s method, the proportion of down-regulation and up-regulation miRNAs were analyzed based on the fold change (2^-ΔΔCq^). It showed that increasing regulations in miRNA 21-5p and miRNA 155-5p (1.74 and 1.05 folds); while in miRNA 29c-3p, there was decreasing regulation of 1.83-fold (Table 1). Based on the fold change analysis, miRNA 29c-3p may be acting as a tumor suppressor, and oppositely, miRNA 21-5p and miRNA 155-5p may act as onco-miR.

**Table 1. Difference in ΔCq, ΔΔCq and fold change of miRNA 29c-3p, miRNA 21-5p, miRNA 155-5p in HCC patients**

**compared with healthy controls**

| **Variables*** | | **HCC (36)** | **Healthy (36)** | | **P^#^** | **Fold change^$^**  **(2^-ΔΔCq^)** | **Fold Regulation** | **Regulation in HCC^¥^** | | |
| --- | --- | --- | --- | --- | --- | --- | --- | --- | --- | --- |
|  |  |  |  |  |  |  |  | **down (%)** | | **up (%)** |
| **mRNA 29c-3p** | **ΔCq _miRNA 29c-3p_** | 6.96 ± 2.63 | 6.09 ± 1.83 | | 0.180 | 0.6 | *Decreasing regulation 1.83 folds* | 19 (52.78) | 17 (47.22) | |
|  | **ΔΔCq _miRNA 29c-3p_** | 0.795 |  |  | | | |  | | |
| **miRNA 21-5p** | **ΔCq _miRNA 21-5p_** | 0.43 ± 2.23 | 1.23 ± 2.16 | | 0.126 | 18.73 | *Increasing regulation 1.74 folds* | 13 (36.11) | 23 (63.8) 9) | |
|  | **ΔΔCq _miRNA 21-5p_** | -3.28 |  |  | | | |  | | |
| **miRNA 155-5p** | **ΔCq _miRNA 155--5p_** | 7.11 ± 1.45 | 7.74 ± 0.94 | | 0.03 | 1.55 | *Increasing regulation 1.55 folds* | 10 (27.78) | 26 (72.22) | |
|  | **ΔΔCq _miRNA 155-5p_** | -0.631 |  |  | | | |  | | |
| The expression of every miRNA was normalized to housekeeping-stably expressed of miRNA-16; HCC: hepatocellular carcinoma  ^*)^analysed by Biorad CFX 96 ManagerTM ; ^#)^ analysed by Mann-Whitney with significant value p<0.05 ; ^$)^Livak’s Method ; ^¥)^ calculated based on value of fold change; HCC: Hepatocellular carcinoma | | | | | | | | | | |

**The relationship between clinical characteristics and miRNA regulation in HCC subjects**

Subjects who had complete based line clinical data and the ΔCq level of miRNA were continuing statistical analyzes. Only 27 HCC patients had completed data.

The characteristic data and clinical laboratory examination showed in Table 2, showing 62.96% of subjects were male with a median of age 55 years old. The increase of AFP ≥200 IU/mL was around 48.15%, with most of the subjects suffering from HBV infection (59.26%) and cirrhosis background (66.67%). 85.18% of subjects were within large tumors >5 cm in diameter and others with multiple tumors (7%). The distribution of BCLC staging C showed that more frequent than B and D. There were only 17 patients who underwent chemotherapy. Trans-arterial chemo embolization (TACE) underwent for 14 patients and sorafenib for three patients only.

The clinical laboratory results showed that the mean of neutrophil, platelet, lymphocyte, and choline esterase within normal limit. The slight decreasing of mean albumin and increasing of mean CRP were showed in the laboratory results at Table 3. There was increasing of mean of NLR (5.94 ± 3.62) based on normal range (1-3), and there was normally of mean PLR based on median normal range (132.4 ± 43.68) [30].

**Table 2.** **The characteristic data for subjects with HCC** **(27 patients)**

| **Variables** | **N (%)** | **Variables** | **N (%)** | **Median**  **(min; max)** |
| --- | --- | --- | --- | --- |
| Sex   - Male - Female | 17 (62.96)  10 (37.04) | Age (y.o) | - | 55 (29;74) |
| Alpha feto protein  (≥ 200 IU/mL) | 13 (48.15) | Cirrhosis | 18 (66.67) | - |
| Hepatitis   - HBV - HCV - None | 16 (59.26)  2 (7.41)  9 (33.33) | BCLC stage  - B  - C  - D | 8 (29.63)  15 (55.56)  4 (14.81) | - |
| Nodule characteristics  - Multiple  - Diameter ≥ 5 cm  - Metastasis  - Thrombus portal vein | 19 (76)  23 (85.18)  5 (18.52)  2 (8.33) | Treatment  - TACE  - Sorafenib | 14 (51.85)  3 (11.11) | - |
| Histories  - Smoking  - Alcohol | 7 (25.93)  3 (11.54) |  |  |  |
| Min: minimum; max: maximum; y.o: year old; BCLC: Barcelona Clinic Liver Cancer; TACE: trans arterial chemoembolization; HCC: Hepatocellular carcinoma | | | | |

**Table 3. The clinical laboratory results (27 HCC patients)**

| **Variables** | **Mean ± SD** | **Median (min; max)** |
| --- | --- | --- |
| Neutrophil (10^3^/µL) | 6.76 ± 2.76 | 6.1 (3.37; 15.41) |
| Lymphocyte (10^3^/µL) | 1.31 ± 0.51 | 1.2 (0.46; 2.61) |
| Platelet (10^3^/µL) | 270.15 ± 111.11 | 243 (118; 722) |
| Albumin (g/dL) | 3.36 ± 0.66 | 3.42 (2.16; 4.4) |
| Choline esterase (U/mL) | 13.26 ± 18.42 | 6.7 (2.8; 77) |
| C-Reactive protein (mg/L) | 44.46 ± 39.40 | 41 (5; 131) |
| Neutrophil-Lymphocyte ratio | 5.94 ± 3.62 | 4.11 (2.32; 15.1) |
| Platelet-Lymphocyte ratio | 234.94 ± 137.71 | 196.03 (91.47; 694.23) |
| SD: standard deviation; min: minimum; max: maximum; HCC: Hepatocellular carcinoma | | |

The difference of miRNAs regulation based on clinical laboratory data showed a significant difference of miRNA 29c-3p regulation with NLR and platelet count (value in down-regulation higher than up-regulation, p<0.05), and also ChE level (value in down-regulation lower then up-regulation, p<0.05). Increasing in NLR and platelet; decreasing of ChE and albumin showed a significant difference in miRNA 29c-3p regulation. Although, in miRNA21-5p and miRNA 155-5p, there was only an increasing neutrophil that showed a significant difference (Table 4).

The OR analyses between miRNAs regulation and clinical variables showed in Table 5. Using cut-off value of NLR ≥4 (AUC= 0.7765, sensitivity 76.47%, and specificity 80% respectively). NLR may be a predictive factor of up-regulation in miRNA 21-5p (OR=3) and miRNA 155-5p (OR=2.63), and even as a protective factor in miRNA 29c-3p (OR=0.11).

Using cut-off value of PLR ≥165.83 (AUC=0.5882; 58.82% sensitivity, and 40% specificity respectively). PLR may be a predictive factor up-regulation of miRNA 29c-3p (OR=1.6) and protective factor up-regulation of miRNA 21-5p (OR=0.65). If cut-off value of PLR used the tertile 1 (T1) (PLR ≥ 292.40). PLR may be a predictive factor up-regulation of miRNA 21-5p (OR=1.62) and miRNA 155-5p (OR=3.11), even PLR in miRNA 29c-3p (OR=0.25) may be changed to be a protective factor.

The minimal normal range of ChE (<7) used as a cut-off value, ChE may be a preventive factor of miRNA 29c-3p and miRNA 21-5p up-regulation (OR=0.44; OR=0.75), even as a predictive factor of miRNA 155-5p up-regulation (OR=2). Based on cut-off albumin value <3.2 showed that albumin might be a predictive factor up-regulation of miRNA 21-5p (OR=3.89), even though as preventive factor up-regulation in miRNA 155-5p (OR=0.55) and miRNA 29c-3p (OR=0.14).

Based on APASL criteria of AFP ≥200 IU/ml, this study results showed AFP might be a predictor factor of up-regulation of miRNA 29c-3p and protective factor for up-regulation of miRNA 21-5p and miRNA 155-5p.

**Table 4. The regulation of miRNA analyses based on clinical variables in 27 HCC patients**

| **Variables** | **miRNA 29c-3p (n)** | | | **miRNA 21-5p (n)** | | | **miRNA 155-5p (n)** | | |
| --- | --- | --- | --- | --- | --- | --- | --- | --- | --- |
|  | **down-reg**  **(9)** | **up-reg**  **(18)** | ***p*^*^** | **down-reg**  **(13)** | **up-reg**  **(14)** | ***p*^*^** | **down-reg**  **(10)** | **up-reg**  **(17)** | ***p*^*^** |
| NLR | 6.84±4.01 | 4.12±1.69 | *0.015* | 6.48±3.90 | 5.44±3.42 | 0.331 | 7.84±4.64 | 4.82±2.37 | 0.175 |
| PLR | 245.53±155.73 | 213.78±96.63 | 0.151 | 244.46±153.32 | 226.1±126.70 | 0.662 | 253.51±108.81 | 224.02±154.32 | 0.228 |
| CRP | 45.55± 41.30 | 42±40.62 | 0.974 | 50.62±41.05 | 34.6±38.87 | 0.462 | 67.33±55.52 | 37.6±34.04 | 0.308 |
| Platelet | 274.95±127.73 | 256.8± 58.29 | *0.018* | 311.31±140.22 | 234.0±56.37 | 0.147 | 286.8±71.36 | 259.16±123.73 | 0.089 |
| ChE | 8.98 ±6.16 | 21.72±30.97 | *<0.001* | 10.25±6.75 | 15.67±24.89 | 0.834 | 8.8±7.05 | 13.92±19.60 | 0.840 |
| Lymphocyte | 1.29±0.54 | 1.36±0.47 | 0.670 | 1.44±0.58 | 1.20±0.42 | 0.497 | 1.29±0.59 | 1.33±0.48 | 0.422 |
| Neutrophil | 7.52±2.93 | 5.22±1.60 | 0.075 | 8.03±3.04 | 5.57±1.87 | *0.017* | 8.47±3.46 | 5.75±1.64 | *0.021* |
| Albumin | 3.29±0.76 | 3.49±0.24 | *0.002* | 3.20±0.74 | 3.49±0.52 | 0.153 | 3.29±0.66 | 3.40±0.64 | 0.792 |
| NLR: Neutrophil-Lymphocyte ratio; PLR: Platelet-Lymphocyte ratio; CRP: C-Reactive protein; ChE: Choline esterase; down-reg: down regulation; up-reg: up regulation; HCC: Hepatocellular carcinoma; reg: regulation  ^*)^ Significant *p*<0.05; using Mann-Whitney test | | | | | | | | | |

**Table 5. The odds ratio analyses of up-regulation miRNA based on cut off value clinical variables in 27 HCC patients**

| **Variables** | **miRNA 29c-3p** | | **miRNA 21-5p** | | **miRNA 155-5p** | |
| --- | --- | --- | --- | --- | --- | --- |
|  | **OR** | **min; max** | **OR** | **min; max** | **OR** | **min; max** |
| NLR≥4 | 0.11 | 0.01; 0.92 | 3 | 0.49;19.82 | 2.63 | 0.4; 20.54 |
| PLR  ≥165.83 | 1.6 | 0.24; 2.89 | 0.65 | 1.10; 3.90 | 1.05 | 0.17;7.11 |
| PLR (T1)  ≥ 292.40 | 0.25 | 0.00; 2.85 | 1.62 | 0.21; 13.96 | 3.11 | 0.37; 27.21 |
| ChE < 7 | 0.44 | 0.03; 6.24 | 0.75 | 0.06; 8.58 | 2 | 0.08; 137.35 |
| Alb<3.2 | 0.14 | 0.00; 1.61 | 3.89 | 0.56; 31.12 | 0.55 | 0.07; 3.75 |
| AFP≥200 | 1.25 | 0.18; 8.87 | 0.32 | 0.45; 2.21 | 0.42 | 0.05; 3.13 |
| NLR: Neutrophil-Lymphocyte ratio; PLR: Platelet-Lymphocyte ratio; PLR (T1): Platelet-Lymphocyte ratio, first tertile value; CRP: C-Reactive protein; ChE: Choline esterase; Alb: albumin; AFP: alpha fetoprotein; OR: odds ratio value; min: minimum; max: maximum | | | | | | |

Insert **Table 6** **Analyses of HCC nodule, BCLC stagging and etiology with miRNAs regulation in 27 HCC subjects**

Insert **Survival analyses of HCC subjects**

**Discussion**

Previous studies mentioned that miRNAs might be detected clearly and are stable in serum/plasma, cancer cells, and cell lines. The regulation of miRNAs indicated that miRNAs have a role in tumor tissue dysregulation as tumor suppressors or oncogenes. The miRNAs can be used as diagnostic, targeting therapy, and prognostic markers in patients with HCC. Some of the miRNAs identified as tumor suppressors or oncogene, with the characteristic indication of poor disease progression [31,32]. The study resulted show that miRNA 29c-3p have a role in tumor-suppressing and miRNA 21-5p, and miRNA 155-5p as tumor oncogenic). Based on Livak’s Method, the decreasing of regulation 1.83-fold in miRNA 29c-3p, increasing regulation 1.74-fold in miRNA 21-5p and 1.55-fold in miRNA 155-5p, respectively. The miRNA 21 (PTEN as a target gene) and miRNA 155 (RhoA and TLR as target genes) have a role in metastasis effects of HCC. Oppositely, miRNA 29c (Bcl-2, Bcl-w, and Ras as target genes) has a role in apoptosis [32].

The circulation of miRNA 21 and miRNA 122 can predict progression LC to HCC with a value of OR >1 [33}. Plasma miRNA 21 and miRNA 122 have been considered potential biomarkers of early liver fibrosis, and they have strong correlations with fibrosis severity in HCV genotype-4. Also, they can be used for early marker detection and diagnosis of HCC if combined with plasma AFP and VEGF [10,34]. Jin et al, 2019 revealed that plasma miRNA 122-5p was one of 5 potential biomarkers in HCC caused by chronic HBV infection [36].

Shen et al. in 2016 revealed that up-regulation of miRNA 21-5p from blood able used as an indicator for diagnosis and prognosis of HCC. The miRNA 21-5p can take the role of diagnosis HCC based on: tumor suppressor and oncomiR, limitless replicative potential, liver injury, and other clinicopathologic features. The miRNA 21-5p can take a role in HCC prognosis based on: limited replicative potential and tissue invasion/metastasis [35]. And also circulating miRNA 21 can serve as a potential co-biomarker in early-stage HCC [36].

The regulation of miRNA 29 from blood and tissue, reported as an oncogene or tumor suppressor, is controversial because of various mechanisms [37]. Down-regulation of miRNA 29c-3p correlated with tumor size, multiplicity pathologic features, and shorter overall survival; and up-regulation of miRNA 29c-3p correlates with inhibition cell proliferation, apoptosis, migration, and tumor growth in vivo [38]. Although, down-regulation of miRNA 29b-3p in blood correlates with tumor suppressor and oncomir only [35].

Besides deregulation of miRNAs, there are discoveries of non-coding RNA (ncRNA) genes related to hepatocarcinogenesis via Wnt and STAT3 signaling pathways, although there are still only limited publications until now [39].

This study examined the relationship between three miRNAs (miRNA 29c-3p, miRNA 21-5p, and miRNA 155-5p) as potential biomarkers of HCC progression, with clinical severity based on routinely done laboratory examinations. There were significant differences in the regulation of miRNA 29c-3p based on NLR, platelet count, ChE, and albumin. Even though, in miRNA 21-5p and miRNA 155-5p, there was only a significant difference in neutrophils (see Table 4).

Based on the OR analyses, NLR≥4 might be a protective factor in up-regulation of miRNA 29c-3p; a predictive factor in up-regulation of miRNA 21-5p and miRNA155-5p (see Table 5). Previous studies mentioned that NRL ≥4 correlated with high mortality among low MELD scores on LC patients because the NRL correlated with pro-inflammatory neutrophils [18]. High NLR might be an independent biomarker prediction of 30-day mortality in HBV-related LC decompensated [40]. Decreasing T lymphocytes results from reducing thymic output and hyperactivation of T lymphocytes. Elevated NLR was positivity correlated with pathogenesis and progression of primary liver cancer related with HBV infection [19].

PLR able to be a reliable prognostic biomarker for liver cancer. Elevated platelets and reduction of lymphocytes were considered indicators of a poor prognosis and can be used to predict the overall survival of liver cancer [20, 41].

This study had some limitations. (1) the observational study using the cross-sectional design; (2) the small study population was conducted; (3) the blood specimen was prepared only for three potential miRNAs (2 up-regulation miRNAs as onco-miR and one down-regulation miRNA as a tumor suppressor). (3) there was no matching between groups in terms of age, gender, and other potential confounding factors.

**Conclusions**

In conclusion, the miRNA 21-5p and miRNA 155-5p might be potential biomarkers as an onco-miR in HCC subjects. The miRNA 29c-3p might be a potential biomarker as a tumor suppressor. A significant result identified in clinical progression based on the regulation of miRNAs, which was consistent with miRNA 29c-3p.

**Acknowledgements**

The authors would like to thank the members of the Internal Medicine Ward and Polyclinic Dr. Sardjito General Hospital, Internal Medicine Department Dr. Margono Soekarjo Hospital, and Cell analysis Laboratory Faculty of Medicine Public Health and Nursing Universitas Gadjah Mada.

The authors also thank the staff of Klinik Bahasa in the Office of Research and Publication of the Faculty of Medicine, Public Health and Nursing, Universitas Gadjah Mada for assistance in language editing and proofreading.

**References**

1. Guan C, Yang F, He X, Li T, Yang Q, He H, et al. Clinical significance of microRNA-155 expression in hepatocellular carcinoma. Oncology Letters. 2016; 11: 1574-1580. [DOI:10.3892/ol.2015.4048]

2. Shariff MIF, Cox IJ, Gamaa AI, Khan SA, Gedroyc W, et al. Hepatocellular carcinoma: current trends in worldwide epidemiology, risk factors, diagnosis and therapeutics. Expert Rev Gastroenterol Hepatol. 2009; 3(4): 353-367. [PMID: 19673623 DOI: 10.1586/egh.09.35]

3. Parkin DM. The global health burden of infection-associated cancers in the year 2002. Int J Cancer. 2006; 118(12): 3030-3044. [PMID: 16404738 DOI: 10.1002/ijc.21731]

4. Gomaa AW, Khan SA, Toledano MB, Waked I, Taylor-Robinson SD. Hepatocellular carcinoma: epidemiology, risk factors and pathogenesis. World J Gastroenterol. 2008; 14(27): 4300-4308. [DOI: 10.3748/wjg.14.4300]

5. Lujambio A, Lowe SW. The microcosmos of cancer. Nature 2012; 482: 347-355. [PMID: 22337054 DOI: 10.1038/nature10888]

6 Carleton M, Cleary MA, Linsley PS. MicroRNAs and cell cycle regulation. Cell Cycle 2007; 6(17): 2127-2132. [DOI: 10.4161/cc.6.17.4641]

7. Szabo G, Sarnow P, Bala S. MicroRNA silencing and the development of novel therapies for liver disease. J Hepatol 2012; 57(2):462-466. [PMID: 22504335 DOI: 10.1016/j.jhep.2012.01.030]

8. Abd-Elmouttaleb AT, Abd-Elatif DM, Soliman GM, Taher MS, Abonar AA. Serum Micro RNA-122 as a biomarker for hepatocellular carcinoma in chronic hepatitis C virus patients. Research in Cancer and Tumor 2015; 4(2): 25- 33. [DOI:10.5923/j.rct.20150402.01]

9. Han JG, Jiang YD, Zhang CH, Yang YM, Pang D, et al. A novel panel of serum miR-21/miR-155/miR-365 as a potential diagnostic biomarker for breast cancer. Ann Surg Treat Res 2017; 92(2): 55-66. [PMID: 28203552 ; DOI: 10.4174/astr.2017.92.2.55]

10. Demerdash HM, Hussien HM, Hassouna E, Arida EA. Detection of microRNA in hepatic cirrhosis and hepatocellular carcinoma in hepatitis C genotype-4 in Egyptian patients. BioMed Res Inter 2017; 10. [PMID: 28642869 DOI: 10.1155/2017/1806069]

11. Hayes CN, Chayama K. MicroRNAs as biomarkers for liver disease and hepatocellular carcinoma. Int J Mol Sci 2016; 17: 280. [PMID: 26927063 DOI: 10.3390/ijms17030280]

12. Bihrer V, Waidmann O., Friedrich-Rust M, Forestier N, Susser S, et al. Serum MicroRNA-21 as Marker for Necroinflammation in Hepatitis C Patients with and without Hepatocellular Carcinoma. PLoS ONE 2011; 6(10): e26971. [DOI: 10.1371/journal.pone.0026971

13. Lestari P, Qoriansas N, Tanjung DS, Fitria MS, Kartika AI, et al. miR-21, miR-29c, and miR-155 as biomarker to develop minimally-invasive diagnostic in hepatocellular carcinoma patient. J Med Sci 2016; 48(4): Supplement. [DOI: 10.19106/JMedScieSup0048042016019]

14. Tang B, Lei B, Qi G, Liang X, Tang F, et al. MicroRNA-155-3p promotes hepatocellular carcinoma formation by suppressing FBXW7 expression. J Exp Clin Cancer Res 2016; 35(93): 12. [DOI: 10.1186/s13046-016-0371-6]

15. Marrero JA, Feng Z, Wang Y, Naguyen MH, Befeler AS, et al. Alpha-fetoprotein, des-gamma carboxyprothrombin, and lectin-bound alpha-fetoprotein in early hepatocellular carcinoma. Gastroenterology 2009; 137: 110-118. [PMID: 19362088 DOI: 10.1053/j.gastro.2009.04.005]

16. European Association for the Study of the Liver. EASL Clinical Practice Guidelines: management of hepatocellular carcinoma. J Hepatol 2018; 69: 182–236. [PMID: 29628281 DOI: 10.1016/j.jhep.2018.03.019]

17. Omata M, Cheng AL, Kokudo N, Kudo M, Lee JM, et al. Asia–Pacific clinical practice guidelines on the management of hepatocellular carcinoma: a 2017 update. Hepatol Int 2017; 11: 317–370. [PMID: 28620797 DOI: 10.1007/s12072-017-9799-90]

18. Kalra A, Wedd JP, Bambha KM, Gralla J, Golden-Mason L, et al. Neutrophil-to-lymphocyte ratio correlates with proinflammatory neutrophils and predicts death in low model for end-stage liver disease patients with cirrhosis. Liver Transpl 2017; 23(2): 155–165. [PMID: 28006875 DOI: 10.1002/lt.24702]

19. Liu X, He L, Han J, Wang L, Li M, et al. Association of neutrophil-lymphocyte ratio and T lymphocytes with the pathogenesis and progression of HBV-associated primary liver cancer. PLoS ONE 2017; 23:12(2). [PMID: 28231294 DOI: 10.1371/journal.pone.0170605]

20. Ma W, Zhang P, Qi J, Gu L, Zang M, et al. Prognostic value of platelet to lymphocyte ratio in hepatocellular carcinoma: a meta-analysis. Sci Rep 2016; 6: 35378 [DOI: 10.1038/srep35378]

21. Garcıa-Ayllon MS, Millan C, Serra-Basante C, Bataller R, Saez-Valero J. Readthrough acetyl cholinesterase is increased in human liver cirrhosis. PLoS ONE 2017; 7(9): e44598. [DOI: 10.1371/journal.pone.0044598]

22. EL Zwahry E, Ahmed DFI, El Masry M, Gad NA. Serum cholinesterase activity helps to distinguish between liver cirrhosis, hepatocellular carcinoma and chronic hepatitis C virus in Egyptian patient. Researcher 2013; 5(7): 33-37.

23. Meng F, Yin X, Ma X, Guo XD, Jin B, et al. Assessment of the value of serum cholinesterase as a liver function test for cirrhotic patients. Biomedical Reports 2013; 1: 265-268. [PMID: 24648933 DOI: 10.3892/br.2013.60]

24. Bayupurnama P, Ratnasari N, Maduseno S, Nurdjanah S. C-reactive protein level in decompensated liver cirrhosis and its correlation with serum alanine aminotransferase and albumin levels. Internet J Gastroenterol 2009; 9(2): 3. [https://print.ispub.com/api/0/ispub-article/8623]

25. Sieghart W, Pinter M, Hucke F, Graziadei I, Schoniger-Hekele M, et al. Single determination of C-reactive protein at the time of diagnosis predicts long-term outcome of patients with hepatocellular carcinoma. Hepatology 2013; 57: 2224-2234. [PMID: 22961713 DOI: 10.1002/hep.26057]

26. Pieria G, Agarwalb B, Burroughsa AK. C-reactive protein and bacterial infection in cirrhosis. Ann Gastroenterol 2014; 27: 113-120. [PMID: 24733601]

27. Chen W, Wang JB, Abnet CC, Dawsey SM, Fan JH, et al. Association between C-reactive protein, incident liver cancer, and chronic liver disease mortality in the Linxian Nutrition Intervention Trials: a nested case–control study. Cancer Epidemiol Biomarkers Prev 2015; 24: 386-392. [PMID: 25613115 DOI: 10.1158/1055-9965]

28. Oh BS, Jang JW, Kwon JH, You CR, Chung KW, et al. Prognostic value of C-reactive protein and neutrophil-to-lymphocyte ratio in patients with hepatocellular carcinoma. BMC Cancer 2013; 13: 78. [PMID: 23409924 DOI: 10.1186/1471-2407-13-78]

29. Livak KJ, Schmittgen TD. Analysis of relative gene expression data using real-time quantitative PCR and. Methods. 2001;25:402-408. [DOI:10.1006/meth.2001.1262]

30. Lee S J, Kim NA, Na SH, Youn YH, Shin CS. Reference values of neutrophil-lymphocyte ratio, lymphocyte-monocyte ratio, platelet-lymphocyte ratio, and mean platelet volume in healthy adults in South Korea. Medicine. 2018; 97 :26 (e11138). [http://dx.doi.org/10.1097/MD.0000000000011138]

31. Mao B and Wang G. MicroRNAs involved with hepatocellular carcinoma (Review). Oncol Rep 2015; 34: 2811-2820. [PMID: 26398882 DOI: 10.3892/or.2015.4275]

32. Hung CH, Chiu YC, Chen CH, Hu TH. MicroRNAs in hepatocellular carcinoma: carcinogenesis, progression, and therapeutic target. BioMed Res Int 2014; 2014: 486407. [PMID: 24800233 DOI: 10.1155/2014/486407]

33. Huang YH, Liang KH, Chien RN, Hu TH, Lin KH, et al. A circulating microRNA signature capable of assessing the risk of hepatocellular carcinoma in cirrhotic patients. Sci Rep 2017; 7: 523. [PMID: 28364124 DOI: 10.1038/s41598-017-00631-9]

34. Abd-Elmouttaleb AT, Abd-Elatif DM, Soliman GM, Taher MS, Abonar AA. Serum micro RNA-122 as a biomarker for hepatocellular carcinoma in chronic hepatitis C virus patients. Research in Cancer and Tumor 2015; 4(2): 25-33. [DOI: 10.5923/j.rct.20150402.01]

35. Shen S, Lin Y, Yuan X, Shen L, Chen J, et al. Biomarker microRNAs for diagnosis, prognosis and treatment of hepatocellular carcinoma: a functional survey and comparison. Sci Rep 2016; 6: 38311. [PMID: 27917899 DOI: 10.1038/srep38311]

36. Liao Q, Han P, Huang Y, Wu Z, Chen Q, et al. Potential Role of Circulating microRNA-21 for Hepatocellular Carcinoma Diagnosis: A Meta-Analysis. PLoS ONE 2015; 10(6): e0130677. [DOI: 10.1371/journal.pone.0130677]

37. Kwon JJ, Factora TD, Dey S, Kota J. A systematic review of miR-29 in cancer. Mol Ther Oncolytics 2019; 12: 173-194. [PMID: 30788428 DOI: 10.1016/j.omto.2018.12.011]

38. Wu H, Zhang W, Wu Z, Liu Y, Shi Y, et al. miR-29c-3p regulates DNMT3B and LATS1 methylation to inhibit tumor progression in hepatocellular carcinoma. Cell Death Dis 2019; 10: 48. [DOI: 10.1038/s41419-018-1281-7]

39. Klingenberg M, Matsuda A, Diederichs S, Patel T. Non-coding RNA in hepatocellular carcinoma: mechanisms, biomarkers and therapeutic targets. J Hepatol 2017; 67: 603-618. [DOI: 10.1016/j.jhep.2017.04.009]

40. Zhang H, Sun Q, Mao W, Fan J, Ye B. Neutrophil-to-lymphocyte ratio predicts early mortality in patients with HBV-related decompensated cirrhosis. Gastroent Res Pract 2016; 5. [PMID: 28438689 DOI: 10.1016/j.jhep.2017.04.009]

41. Huang GQ, Zheng JN, Zouz TT, Chen YR, Shi KQ, et al. Stratified platelet-to-lymphocyte ratio: a novel target for prognostic prediction of hepatocellular carcinoma after curative liver resection. J Clin Transl Hepatol 2017; 5: 35–42. [DOI: 10.14218/JCTH.2016.00035]
